# Bidirectional intergenerational support and mental health in older adults based on latent profile analysis: a moderated mediation model

**DOI:** 10.3389/fpubh.2025.1685701

**Published:** 2025-12-10

**Authors:** Ao Li, Xiaoxi Xie, Xinyang Wan, Zhanjing Dai, Feng Chang, Yun Lu

**Affiliations:** School of International Pharmaceutical Business, China Pharmaceutical University, Nanjing, Jiangsu, China

**Keywords:** older adults, intergenerational support, mental health, latent profile analysis, social participation

## Abstract

**Background:**

Bidirectional intergenerational support is linked to late-life mental health, yet the underlying mechanisms remain unclear. Guided by intergenerational solidarity and social support theories, we examined how distinct support profiles relate to mental health among Chinese older adults, testing self-rated health (SRH) as a mediator and social participation as a moderator.

**Methods:**

We analyzed 7,843 adults aged ≥60 from the 2020 China Longitudinal Aging Social Survey. Latent profile analysis (LPA) identified bidirectional support profiles; group differences in mental health were assessed using the Bolck-Croon-Hagenaars (BCH) approach, followed by mediation and moderated-mediation models with bootstrap inference (5,000 resamples).

**Results:**

Four profiles emerged—High Support-High Interaction-High Closeness (HS-HI-HC; 47.02%), Child-High Support-Low Interaction-High Closeness (CS-LI-HC; 33.46%), Moderate Support-Moderate Interaction-Low Closeness (MS-MI-LC; 10.37%), and Low Support-Low Interaction-Moderate Closeness (LS-LI-MC; 9.16%). Mental health differed across different profiles, with HS-HI-HC showing the best mental health levels (the lowest scores). SRH partially mediated these associations (for instance, HS-HI-HC indirect effect = −0.186, 95% CI -0.245 to −0.131). Social participation attenuated benefits of high family support but buffered risks under low support.

**Conclusion:**

Bidirectional intergenerational support is heterogeneous in China; profiles characterized by reciprocity and closeness show the most favorable mental health. SRH accounts for a modest but meaningful share of these associations, and social participation can substitute for—or amplify—the benefits of family support depending on profile. Findings inform profile-tailored community and family interventions to promote healthy aging.

## 1 Introduction

With advancements in the economy and healthcare, life expectancy has steadily increased, while fertility rates have declined due to shifts in reproductive preferences. Consequently, the proportion of middle-aged and older adults has risen, exacerbating population aging. According to the World Health Organization, by 2030, one in six people globally will be aged 60 or above, and by 2050, 80% of older adults will reside in low and middle income countries. China’s 2021 national census reported 280 million people aged 60+, comprising 19.8% of the population—an increase of 6.6 percentage points since 2012. This demographic shift poses substantial challenges to economic growth, social security, and healthcare systems (1). Notably, mental health problems among older adults have become increasingly prominent. The WHO reports that approximately 14% of older individuals suffer from a mental health disorder, with depression and anxiety being most prevalent. Chinese studies further indicate generally low levels of psychological well-being among elders, driven by factors such as loneliness, inadequate social support, and chronic diseases (2). These mental health issues compromise quality of life and impose additional burdens on families and society. As China’s aging intensifies, scholarly attention to the mental health of older adults is growing accordingly.

In contemporary China, the older adults care system remains predominantly family-based, with informal caregiving within the household continuing to serve as the primary mode of support for older adults. As a traditional means of elder care, intergenerational support plays a vital role in the lives of older adults across diverse cultural settings and exerts a sustained influence on their psychological well-being (3). In Chinese family culture, intergenerational relationships are rooted in long-standing Confucian norms that emphasize filial piety and reciprocal obligations between adult children and their parents. These norms require adult children to provide economic support, caregiving, and emotional support, while older people often repay their children by looking after their grandchildren and helping with household chores. Earlier scholarship often framed intergenerational support as a unidirectional flow of resources, primarily emphasizing children’s obligations to provide for their aging parents (4). In recent years, however, with accelerating urbanization and modernization, intergenerational support has increasingly been conceptualized as a bidirectional process (5, 6). Older adults not only receive support from their children but also contribute to their children’s well-being through grandchild care, household assistance, and other means (7). Such reciprocal relationships may enhance older adults’ sense of self-worth and social engagement, thereby positively influencing their mental health (8). Intergenerational solidarity theory provides a useful theoretical lens for understanding the relationship between bidirectional intergenerational support and older adults’ mental health (9, 10). The theory posits that family intergenerational relations are composed of multiple dimensions, including structural, affective, associational, normative, functional, and consensual solidarity (11). These varying dimensions of intergenerational ties may exert differential effects on the well-being of older adults (12). Empirical research has demonstrated the positive impact of bidirectional intergenerational support on older adults’ psychological well-being. Economic, caregiving, and emotional support from children have been found to significantly enhance older adults’ life satisfaction and mental health (13, 14). Similarly, when older adults provide care or assistance to their children, they may experience a strengthened sense of self-worth, which in turn promotes better psychological outcomes (15, 16). However, the effects of bidirectional support are not uniformly beneficial. If older adults continuously provide excessive care and support to their children, this can lead to increased psychological strain and adverse mental health outcomes (10, 17). Latent class analyses have revealed considerable regional variation in intergenerational support patterns across China (18–22). However, few studies to date have examined how different patterns of bidirectional intergenerational support affect the mental health of older adults.

Self-rated health (SRH) is an individual’s subjective evaluation of their own health status and has become a widely used indicator in health research, particularly in studies of older adults, such as those predicting health-related outcomes and the use of healthcare services (23–27). Older adults with poor SRH are more likely to experience symptoms of depression and anxiety; (28–30) as physical health deteriorates, psychological well-being often declines accordingly (31, 32). Positive self-rated health not only reflects an individual’s satisfaction with their physical condition but may also improve mental health by enhancing one’s sense of control over life and fostering a more optimistic outlook (30, 33). Theoretically, SRH functions as a key mediator linking intergenerational support to mental health. Drawing from intergenerational solidarity theory, the functional (for instance, caregiving, economic support) provided by children directly improves older adults’ economic well-being and access to medical services which enhances an older adult’s capacity to maintain their health and emotional support exchanged with family fosters a sense of being cared for (34). This provision of resources and affective affirmation is hypothesized to first translate into a more positive subjective assessment of one’s own health (SRH). In turn, positive SRH directly contributes to better mental health outcomes. Thus, SRH is a plausible pathway through which the benefits of intergenerational solidarity are realized as mental well-being. However, to date, little research has explored the mediating role of self-rated health in the relationship between bidirectional intergenerational support and mental health among older adults.

According to social support theory, active participation in social activities helps individuals expand their social networks and access more external resources, thereby alleviating psychological stress and negative emotions, and ultimately improving mental health (35–38). Among older adults, social participation is defined as meaningful engagement in social and productive activities, or involvement in activities that entail personal agency and contribute to others. Such participation includes community engagement, volunteering, and recreational or social events (39). Prior studies have confirmed that higher levels of social participation are significantly associated with better mental health and improved self-rated health among older adults (40–44). Drawing from social support theory, social participation is conceptualized as a key moderator. Social support theory posits that individuals draw resources from multiple networks, including family and the wider community. Intergenerational support represents the primary family network, while social participation represents a important community network. According to the buffering hypothesis, these sources can act as substitutes. For instance, high levels of social participation can offer emotional compensation and material resources when intergenerational support is lacking, thus mitigating the psychological and health risks associated with insufficient family-based support (45). In contrast, older adults with limited social participation may rely more heavily on family-based intergenerational support to maintain their psychological well-being (46). This theoretical mechanism justifies its role as a moderator. Therefore, the level of social participation may moderate both the direct relationship between bidirectional intergenerational support profiles and mental health, as well as the indirect pathway through self-rated health.

**Figure 1:**
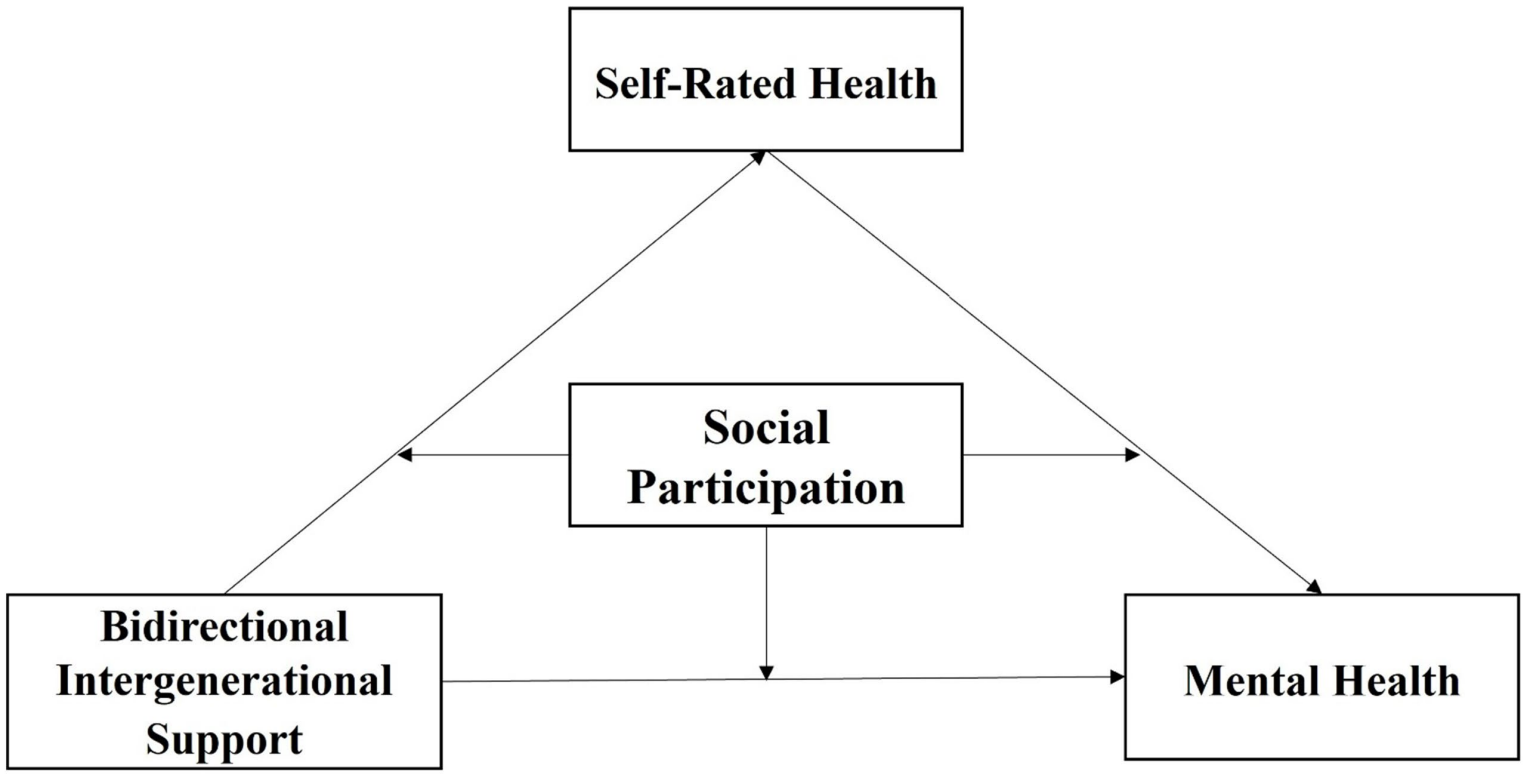
Conceptual framework of hypothesized relationships.

Existing research has primarily focused on the classification of intergenerational support profiles and the direct associations between intergenerational support, self-rated health, or social participation and the mental health of older adults. However, relatively few studies have examined how different profiles of bidirectional intergenerational support influence mental health, the mediating role of self-rated health in this relationship, and under what conditions social participation may amplify or attenuate these effects. Robust empirical evidence addressing these questions remains limited. In response, this study aims to analyze the profiles of bidirectional intergenerational support among Chinese older adults and investigate the underlying mechanisms through which these support profiles affect mental health. Specifically, it examines the mediating role of self-rated health and the moderating role of social participation. This study seeks to deepen theoretical understanding of the mechanisms linking bidirectional intergenerational support and mental health in later life. In doing so, it also highlights the importance of self-rated health and social participation as key psychological and behavioral pathways. Furthermore, the findings aim to inform evidence-based policy interventions that contribute to active and healthy aging in the face of rapid population aging. Based on the literature review and the intergenerational solidarity and social support theories, a conceptual framework was developed to guide this study (Figure 1), which presents the conceptual model linking bidirectional intergenerational support profiles to mental health via direct and indirect pathways and anchors Hypotheses H1-H4.

*H*1: Bidirectional intergenerational support profiles have a significant effect on mental health in older adults.

*H*2: The impact of different intergenerational support profiles varies on mental health in older adults.

*H*3: Self-rated health mediates the relationship between intergenerational support profiles and mental health.

*H*4: Social participation moderates the relationships among intergenerational support profiles, self-rated health, and mental health in older adults.

## 2 Method

### 2.1 Data source

**Figure 2:**
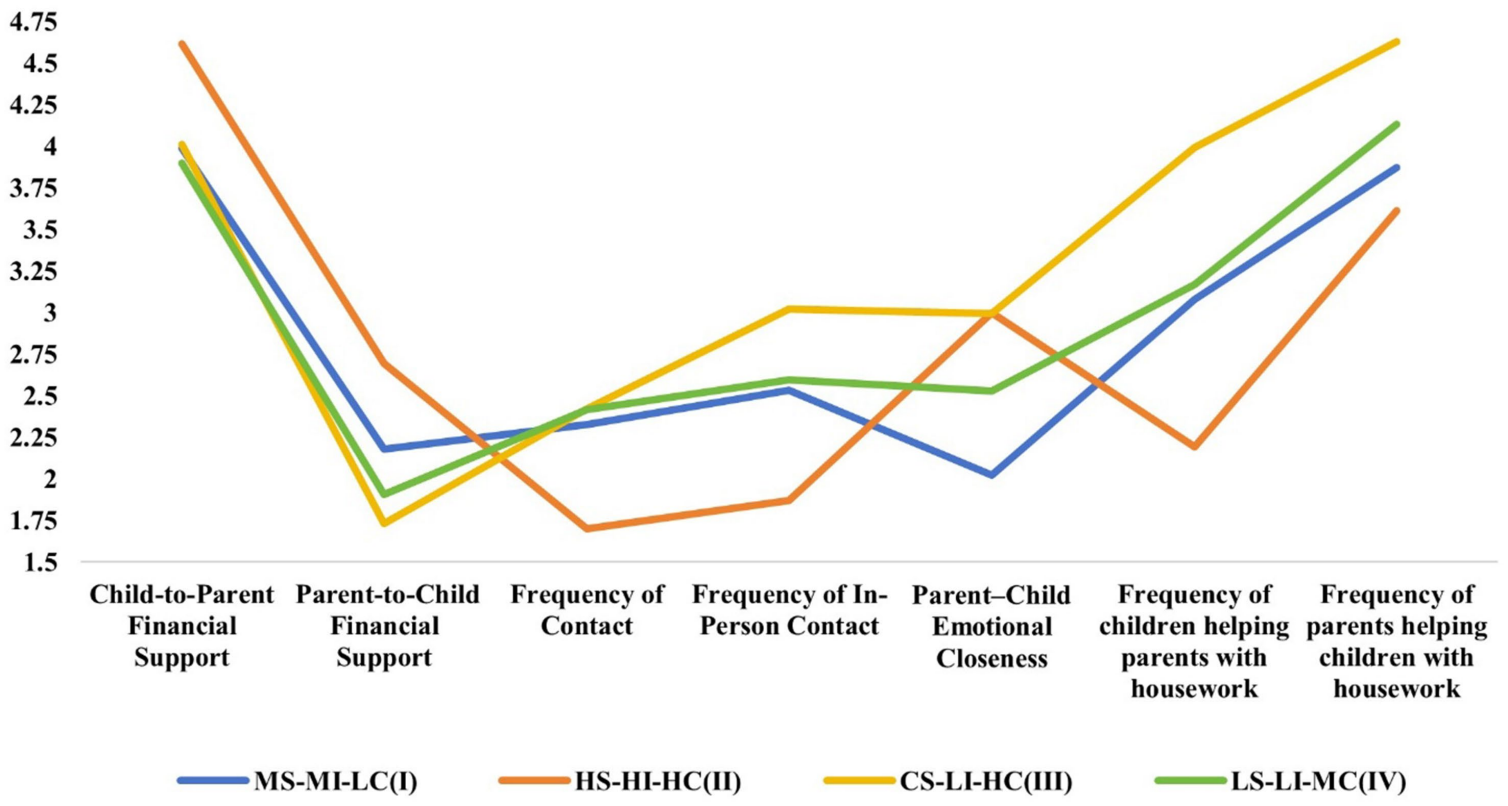
Four latent profiles of bidirectional intergenerational support. This plot shows (unstandardized) means for each profile. For “Frequency of Contact,” “Frequency of In-Person Contact,” “Frequency of children helping parents with housework,” and “Frequency of parents helping children with housework,” higher scores indicate less frequent interaction or help. For all other indicators, higher scores indicate more support or closeness (see Table 2 for details).

The data used in this study were derived from the 2020 wave of the China Longitudinal Aging Social Survey (CLASS), a nationally representative and ongoing survey of Chinese older adults. CLASS employed a stratified multistage probability sampling design, covering 462 villages and urban neighborhoods across 28 provinces, autonomous regions, and municipalities (47). The CLASS survey was conducted using in-home, face-to-face interviews. Trained enumerators administered the questionnaire by reading each question to the participants and recording their verbal responses, which ensured the valid inclusion of all participants. Eligibility for the present analyses was age ≥60 years with valid responses on the key variables of intergenerational support indicators, mental health, self-rated health, and social participation. Following data cleaning procedures that involved removing observations with missing values on key variables or implausible responses (48), the final analytical sample included 7,843 individuals aged 60 and older (Figure 2).

### 2.2 Variable measurement

Dependent Variable: Mental health was measured using the 9-item revised version of the Center for Epidemiologic Studies Depression Scale (CES-D) included in the CLASS questionnaire, which has been widely validated in Chinese older populations (49, 50). The scale consists of six negative and three positive affect items, each rated on a three-point Likert scale ranging from 1 (never) to 3 (often). Positive items were reverse-coded. Total scores range from 9 to 27, with higher scores indicating greater depressive symptomatology and, thus, poorer mental health (21, 28, 51, 52). In this study, the Cronbach coefficient of the revised CESD scale was 0.67, which was considered acceptable, and the KMO was 0.79, indicating good structural validity (53).

Independent Variables: Based on prior literature, this study conceptualized bidirectional intergenerational support using three dimensions: economic, emotional, and caregiving support. These were operationalized using indicators extracted from the “Family and Children” module of the CLASS survey, which captures information on up to 10 children per respondent. Mean values across children were computed to generate continuous variables for each dimension.

Economic Support: Two items measured the frequency of economic transfers in both directions—children to parents and parents to children—over the past 12 months. Each item was rated on a 10-point scale ranging from 1 (no support) to 10 (RMB 2,000 or more), with higher scores reflecting more substantial economic support.

Emotional Support: Three items assessed contact frequency, meeting frequency, and perceived closeness between the respondents and their children. Contact and meeting frequencies were scored on a 5-point scale from 1 (almost daily) to 5 (rarely), with higher scores denoting lower interaction frequency. Closeness was rated from 1 (not close) to 3 (very close), with higher values indicating stronger emotional bonds.

Caregiving Support: This dimension included two items: how frequently children assisted their parents and how frequently parents helped their children with housework over the past year. Both items were scored on a 5-point scale from 1 (almost daily) to 5 (rarely), with higher scores indicating lower levels of caregiving exchange.

Mediating Variable: Self-rated health was measured using a single item from the CLASS questionnaire: “How would you rate your current physical health?” Responses ranged from 1 (very healthy) to 5 (very unhealthy), with higher scores indicating poorer perceived health status (54, 55). Although single-item, SRH has well-documented predictive validity for morbidity and mortality in older adults and is widely used in gerontological research (56), supporting its use as a mediator in this context.

Moderating Variable: Social participation was measured through engagement in three types of activities: economic, political, and public-interest-related (41). Economic participation was assessed based on whether respondents had engaged in paid work or income-generating activities in the past year (0 = no, 1 = yes). Political participation referred to involvement in local residential or village committee elections in the past 3 years (0 = no, 1 = yes). Public-interest participation was measured by whether respondents had taken part in formal volunteering or informal helping behaviors (e.g., neighborhood patrols or community mediation) in the past year (0 = no, 1 = yes). A composite index was created by summing the three indicators, yielding a score ranging from 0 to 3, with higher scores indicating greater social participation. Given its formative nature, we treat it as an index rather than a reflective scale (41).

Control Variables: To account for potential confounding, this study included a set of control variables across three domains: individual characteristics, socioeconomic status, and health conditions. Individual characteristics included gender (0 = female, 1 = male), place of residence (0 = urban, 1 = rural), age (1 = 60–69 years, 2 = 70–79 years, 3 = 80 years and above), marital status (1 = married, 2 = widowed, 3 = divorced, 4 = never married), and educational attainment (ranging from 1 = illiterate to 7 = bachelor degree or above). Socioeconomic status was assessed using log-transformed monthly household expenditures (as a proxy for income), the number of social security programs received, living arrangement (0 = co-residing, 1 = living alone), and the number of surviving children. Health conditions included disability status (0 = no disability, 1 = disabled) and the chronic status is measured by asking older adults the number of chronic diseases they have, and a total of 23 categories of chronic diseases are covered (0–23).

### 2.3 Data analysis

Data were first cleaned and screened using SPSS 27.0, with listwise deletion employed for handling missing values. Descriptive statistics and correlation analyses were then conducted. Next, latent profile analysis (LPA) was performed using Mplus 8.3 to classify bidirectional intergenerational support profiles and examine their distributions and associations with other study variables. The analysis proceeded in several stages: First, LPA was used to identify the optimal number of support profiles based on model fit indices. Second, using the BCH method, we tested whether significant differences in mental health existed across identified profiles. Third, mediation analysis was conducted using SPSS PROCESS 4.0 and a bootstrapping procedure (5,000 resamples) to test whether self-rated health mediated the relationship between support profiles and mental health. Finally, moderated mediation analysis was performed with social participation as a moderator, using the same SPSS PROCESS 4.0 to test for moderated indirect effects. For the moderated mediation analysis, the continuous mediator (SRH) and moderator (Social Participation) were mean-centered before creating interaction terms, as is standard practice. For all regression models involving the latent profiles as a predictor, the MS-MI-LC (I) profile was consistently used as the reference profile.

## 3 Results

### 3.1 Descriptive statistics

This study included a total of 7,843 older adults. Regarding sociodemographic characteristics, 50.1% were female and 49.9% were male; ages were concentrated in the 60–69 (45.0%) and 70–79 (41.6%) age groups, with only 13.3% aged 80 or above. Marital status was predominantly “Married” (76.8%), followed by “Widowed” (22.3%), with other marital statuses being relatively rare. Educational attainment was mainly “Elementary School” (37.1%) and “Junior High” (25.3%), while 22.2% were illiterate. In terms of place of residence, 56.6% was urban and 43.4% was rural. For socioeconomic and health conditions, the mean of the logarithm of income was 7.41 (SD = 0.98), the average number of surviving children was 2.42 (SD = 1.24), and a high proportion (90.6%) were not living alone. The mean number of chronic diseases was 1.69 (SD = 1.39), and most were not disabled (94.1%). In addition, respondents received an average of 1.50 types of social security programs (SD = 0.67).

As for the core variables, the mean scores for mental health and self-rated health (both with higher scores indicating poorer health) were 15.73 (SD = 3.23) and 2.58 (SD = 0.88), respectively; social participation (with higher scores indicating greater participation) averaged 0.98 (SD = 0.85). Regarding bidirectional intergenerational support, the mean scores for children-to-parent financial support and parent-to-children financial support (both with higher scores indicating more support) were 4.28 (SD = 1.66) and 2.24 (SD = 1.64), respectively. For interaction indicators (higher scores indicating lower frequency), the mean contact frequency was 2.08 (SD = 0.70) and the mean meeting frequency was 2.40 (SD = 0.88). Emotional closeness (higher scores indicating greater closeness) averaged 2.85 (SD = 0.32). In terms of household support (higher scores indicating less frequent help), the mean for children helping parents with housework was 2.99 (SD = 1.20) and for parents helping children with housework was 4.03 (SD = 1.29; Table 1). Pairwise associations among variables are reported in the correlation matrix, which is provided in Supplementary Table S1.

**Table 1:** Descriptive statistics for the total sample.

Variables		Percentage/mean	SD	Variable definition/value range
Child-to-parent economic support		4.28	1.66	1–10
Parent-to-child economic support		2.24	1.64	1–10
Frequency of contact		2.08	0.70	1–5
Frequency of in-person contact		2.40	0.88	1–5
Parent–child emotional closeness		2.85	0.32	1–3
Frequency of children helping parents with housework		2.99	1.20	1–5
Frequency of parents helping children with housework		4.03	1.29	1–5
Age	60–69 years old	45.00	0.70	60–69 Years Old = 1
	70–79 years old	41.60	0.70	70–79 Years Old = 2
	80 years of age and older	13.30	0.70	80 years of age and older = 3
Gender	Female	50.10	0.50	Female = 0
	Male	49.90	0.50	Male = 1
Marital status	Married	76.80	0.45	Married = 1
	Widowed	22.30	0.45	Widowed = 2
	Divorced	0.80	0.45	Divorced = 3
	Never married	0.00	0.45	never married = 4
Educational attainment	Illiterate	22.20	1.34	Illiterate = 1
	Private school/literacy class	4.00	1.34	Private School/literacy Class = 2
	Elementary school	37.10	1.34	Elementary School = 3
	Junior high	25.30	1.34	Junior High = 4
	Senior high/technical secondary school	9.00	1.34	Senior High/Technical Secondary School = 5
	Junior college	2.00	1.34	Junior College = 6
	Bachelor’s degree or above	0.50	1.34	Bachelor’s Degree or Above = 7
Income		7.41	0.98	3–11.51
Number of surviving children		2.42	1.24	1–9
Number of social security programs		1.50	0.67	0–8
Living arrangement	Co-residing	90.60	0.29	Co-residing = 0
	Living alone	9.40	0.29	Living Alone = 1
Chronic status		1.69	1.39	0–23
Disability status	No disability	94.10	0.24	No Disability = 0
	Disabled	5.90	0.24	Disabled = 1
SRH		2.58	0.88	1–5
Social participation		0.98	0.85	0–3
Place of residence	Urban	56.60	0.50	Urban = 0
	Rural	43.40	0.50	Rural = 1
Mental health		15.73	3.23	9–27

### 3.2 Latent profile analysis results

Table 2 shows the indicators used in Latent Profile Analysis (LPA). This study utilized Mplus 8.3 to conduct a latent profile analysis of bidirectional intergenerational support, with the latent profiles of bidirectional intergenerational support sequentially set as Profile I, Profile II, Profile III, Profile IV, and Profile V (Table 3). Generally, the smaller the values of AIC, BIC, and ABIC, the closer the Entropy value is to 1, and when LMR and BLRT reach a significant level, it indicated that the model fits better and was considered the optimal choice. The results showed that in the bidirectional intergenerational support group, the significant values of the LMR-LPR and BLRT indicators were simultaneously present in Profiles II, III, IV, and V models, and the Entropy values of the II, III, IV, and V models were above 0.8, indicating that all models had high classification accuracy. Information criteria continued to decline through 5 profiles; however, the additional reduction from 4 to 5 profiles was much smaller than from 3 to 4 (slope ratio = 0.418), contributing only 7.89% of the total BIC improvement from 1 to 5. Meanwhile, entropy decreased from 0.895 to 0.873. Therefore, compared to the II, III, and V models, the values of AIC, BIC, and ABIC for the IV model were kept as small as possible while the Entropy value was as large as possible. Finally, this study comprehensively considered all indicators and selected the IV model as the latent profile result for bidirectional intergenerational support. Additionally, the selected profile model met the criteria proposed by Nagin, where the proportion of individuals in each profile exceeds 5% of the total sample (57), as well as the expectations and assumptions regarding the number of profiles in previous studies (19).

**Table 2:** Indicators used in latent profile analysis.

Dimension	Indicator	Original scale	Direction
Economic	Child-to-parent economic support	1 (no support)–10 (RMB 2,000 or more)	Higher Scores Reflect More Support
	Parent-to-child economic support	1 (no support)–10 (RMB 2,000 or more)	Higher Scores Reflect More Support
Emotional	Frequency of contact	1 (almost daily)–5 (rarely)	Higher Scores Reflect Less Interaction Frequency
	Frequency of in-person contact	1 (almost daily)–5 (rarely)	Higher Scores Reflect Less Interaction Frequency
	Parent–child emotional closeness	1 (not close)–3 (very close)	Higher Scores Reflect More Closeness
Caregiving	Frequency of children helping parents with housework	1 (almost daily)–5 (rarely)	Higher Scores Reflect Less Caregiving
	Frequency of parents helping children with housework	1 (almost daily)–5 (rarely)	Higher Scores Reflect Less Caregiving

**Table 3:** Fit statistics for latent profile models of bidirectional intergenerational support.

Profile	AIC	BIC	aBIC	LMR(p)	BLRT(p)	Entropy	Proportions
I	152672.030	152769.573	152725.084	——	——	——	——
II	142049.657	142202.939	142133.027	0.000	0.000	0.993	0.83973/0.16027
III	132746.598	132955.620	132860.286	0.000	0.000	0.995	0.80377/0.10366/0.09257
IV	127585.541	127850.302	127729.545	0.000	0.000	0.895	0.10366/0.47023/0.33457/0.09155
V	125396.345	125716.844	125570.665	0.000	0.000	0.873	0.18768/0.10366/0.09078/0.31264/0.30524

*p < 0.05, **p < 0.01, ***p < 0.001.

Based on the results of the IV model, this study concluded that there are four latent profiles of bidirectional intergenerational support in the sample group of older adults, of which there were 813 people in the first profile, accounting for 10.37%; 3,688 people in the second profile, accounting for 47.02%; 2,624 people in the third profile, accounting for 33.46%; and 718 people in the fourth profile, accounting for 9.16%. Based on the indicators of economic support, emotional support and caregiving support, this study named the four profiles: in the first profile the economic support and caregiving support between children and their parents were at the moderate level, the frequency of contact and meeting was on the low side, which indicated that the frequency of contact and meeting is more frequent, but the closeness was on the low side, which indicated that older adults have the lowest degree of closeness to their children. Therefore, it was named Moderate Support-Moderate Interaction-Low Closeness (MS-MI-LC); in the second profile, the economic support and care support, contact and meeting frequency, and the degree of intimacy between children and parents were all at the highest level, so it was named High Support-High Interaction-High Closeness (HS-HI-HC); in the third profile, the economic support from children to parents was at the moderate level, indicating that contact and meeting frequency was more frequent, but the degree of intimacy was the lowest, indicating that older adults have the lowest degree of intimacy with their children. The degree of closeness was the second highest, which indicated that older adults are close to their children; the value of mutual help in household chores between the children and their parents was the highest, which reflected that mutual help in household chores is the least frequent, and the level of support for caregiving was the lowest, therefore, it was named Child-High Support-Low Interaction-High Closeness (CS-LI-HC); in the fourth profile, the economic and caregiving support, contact and meeting frequency, and closeness between children and parents were all at a lower level, so it was named Low Support-Low Interaction-Moderate Closeness (LS-LI-MC).

It is worth noting that among the four profiles, only a small number of older adults were categorized as LS-LI-MC (9.16%) or MS-MI-LC (10.37%), while the majority of older adults were categorized as HS-HI-HC (47.02%) or CS-LI-HC (33.46%), which suggested that the overall level of bidirectional intergenerational support was high.

ANOVA found that HS-HI-HC was significantly better than the other three profiles on all three dimensions; LS-LI-MC was at a lower level overall on all three dimensions and was in the third position; MS-MI-LC was at an intermediate level overall on all three dimensions but the closeness was the lowest; and CS-LI-HC was at a low overall level on all three dimensions but had a high degree of closeness. This suggested that there was heterogeneity across different latent profiles (Table 4).

**Table 4:** Mean differences in support indicators across bidirectional intergenerational support profiles.

Profile	Child-to-parent economic support	Parent-to-child economic support	Frequency of contact	Frequency of in-person contact	Parent–child emotional closeness	Frequency of children helping parents with housework	Frequency of parents helping children with housework
MS-MI-LC (I)	3.9908 (1.745)	2.1816 (1.60492)	2.3269 (0.74463)	2.5353 (0.99712)	2.0232 (0.07105)	3.0801 (1.20044)	3.8739 (1.37498)
HS-HI-HC (II)	4.6167 (1.66453)	2.7032 (1.79517)	1.6951 (0.51639)	1.8589 (0.51512)	2.9986 (0.01799)	2.1681 (0.7274)	3.609 (1.43511)
CS-LI-HC (III)	4.0016 (1.60347)	1.7049 (1.2825)	2.4396 (0.6446)	3.0616 (0.7793)	2.9968 (0.02547)	4.057 (0.84514)	4.6521 (0.73127)
LS-LI-MC (IV)	3.9015 (1.32425)	1.8993 (1.28483)	2.4173 (0.66169)	2.5965 (0.75897)	2.5312 (0.13001)	3.17 (1.01658)	4.1398 (1.06587)
F	100.079***	218.589***	936.41***	1564.965***	104810.253***	2509.484***	390.998***
LSD	4 < 1 < 3 < 2	3 < 4 < 1 < 2	2 < 1 < 4 < 3	2 < 1 < 4 < 3	1 < 4 < 3 < 2	2 < 1 < 4 < 3	2 < 1 < 4 < 3

*p < 0.05, **p < 0.01, ***p < 0.001.

### 3.3 Effects of bidirectional intergenerational support profiles on mental health in older adults

Based on the four finalized latent profiles of bidirectional intergenerational support, this study used the BCH method to analyze differences in mental health among older adults across profiles. Results from the omnibus chi-square test indicated significant differences in the effects of different bidirectional intergenerational support profiles on mental health, supporting H1. The mean score ranking was as follows: I > IV > III > II. Mental health differed significantly between all profile groups, and the average mental health score for Profile I was significantly higher than that of the other three groups, indicating that older adults in this profile had the lowest level of mental health, confirming H2 (Table 5).

**Table 5:** Between-group differences in mental health across intergenerational support profiles.

Variable	Profile	Mean	S. E.	Between-group differences(class k-class k + i, i = 0, 1, 2, 3)				Omnibus chi-square
				1	2	3	4	
Mental health	MS-MI-LC (I)	17.148	0.099	0	—	—	—	264.083***
	HS-HI-HC (II)	15.331	0.06	247.456***	0	—	—	
	CS-LI-HC (III)	15.735	0.066	142.186***	17.827***	0	—	
	LS-LI-MC (IV)	16.182	0.111	42.523***	45.39***	11.925***	0	

*p < 0.05, **p < 0.01, ***p < 0.001.

### 3.4 Mediation analysis: self-rated health as a mediator between bidirectional intergenerational support profiles and mental health

After controlling for individual characteristics, socioeconomic status, and health conditions of older adults, bidirectional intergenerational support was used as the independent variable, self-rated health as the mediating variable, and mental health as the dependent variable. A bootstrap method with 5,000 resamples was employed to test the mediation effects of the multicategory independent variable, where bidirectional intergenerational support was a four-category variable derived from the latent profile analysis results.

The results showed that, compared with the MS-MI-LC, the HS-HI-HC, the CS-LI-HC, and the LS-LI-MC had significant positive predictive effects on older adults’ mental health. After including the mediating variable of self-rated health, the direct predictive effects of the HS-HI-HC, the CS-LI-HC, and the LS-LI-MC on mental health remained significant, while self-rated health significantly and positively predicted mental health (Table 6).

**Table 6:** Mediation model results (*n* = 7,843).

Variables	Model 1(dependent variable: mental health)		Model 2(dependent variable: SRH)		Model 3(dependent variable: mental health)	
	β(Standardizedβ)	t	β(Standardizedβ)	t	β(Standardizedβ)	t
HS-HI-HC (II)	−1.7449(−0.5403)	−14.4437***	−0.2349(−0.2676)	−7.2974***	−1.5589(−0.4827)	−13.1557***
CS-LI-HC (III)	−1.3145(−0.4070)	−10.4385***	−0.1079(−0.1229)	−3.2148**	−1.229(−0.3806)	−9.9777***
LS-LI-MC (IV)	−1.1853(−0.3671)	−7.3521***	−0.1459(−0.1662)	−3.3953***	−1.0698(−0.3313)	−6.7831***
Age	0.2472 (0.0532)	3.5708***	0.0826 (0.0654)	4.4746***	0.1819 (0.0391)	2.6836**
Gender	−0.2252(−0.0349)	−3.1097**	−0.036(−0.0205)	−1.8653	−0.1967(−0.0305)	−2.7778**
Marital status	0.2751 (0.0381)	2.8289**	0.0995 (0.0507)	3.8396***	0.1963 (0.0272)	2.0632*
Educational attainment	−0.2088(−0.0866)	−6.9777***	−0.0327(−0.0499)	−4.1032***	−0.1829(−0.0759)	−6.2456***
Income	0.1892 (0.0572)	4.3534***	0.0166 (0.0185)	1.4354	0.176 (0.0532)	4.1430***
Number of surviving children	−0.1507(−0.0576)	−4.5134***	0.0002 (0.0003)	0.0267	−0.1509(−0.0577)	−4.6228***
Number of social security programs	0.1888 (0.0390)	2.8516**	−0.003(−0.0022)	−0.1675	0.1912 (0.0394)	2.9533**
Living arrangement	0.2496 (0.0226)	1.6903	−0.0445(−0.0148)	−1.1300	0.2849 (0.0257)	1.9729*
Chronic status	0.2048 (0.0884)	7.7888***	0.154 (0.2446)	21.983***	0.0828 (0.0358)	3.1277**
Disability status	0.9952 (0.0726)	6.5067***	0.4824 (0.1294)	11.8354***	0.6132 (0.0447)	4.0654***
Place of residence	0.7221 (0.1109)	8.7467***	−0.0146(−0.0082)	−0.6629	0.7337 (0.1126)	9.0904***
SRH	—	—	—	—	0.7918 (0.2152)	19.0968***
R2	0.0840		0.1198		0.1248	
F	51.2720***		76.0984***		74.3898***	

Reference profile is MS-MI-LC; *p < 0.05, **p < 0.01, ***p < 0.001.

The relative mediation analysis showed that, using the MS-MI-LC as the reference, the 95% CI for the relative mediation effect of the HS-HI-HC was [−0.2443, −0.1294], excluding zero, indicating a significant relative mediation effect (a₁ = −0.2349, b = 0.7918, a₁b = −0.1860). This meant that older adults in the HS-HI-HC had self-rated health scores 0.2349 points lower than those in the MS-MI-LC (a₁ = −0.2349). The relative direct effect was significant (c′₁ = −1.5589, *p* < 0.001), indicating that after excluding the mediating effect, older adults in the HS-HI-HC still had mental health scores 1.5589 points lower than those in MS-MI-LC. The relative total effect was significant (c₁ = −1.7449, *p* < 0.001), with the effect size of the relative mediation effect (a₁b) being 10.66%.

Using the MS-MI-LC as the reference, the 95% CI for the relative mediation effect of the CS-LI-HC was [−0.1413, −0.0310], excluding zero, indicating a significant relative mediation effect (a₁ = −0.1079, b = 0.7918, a₁b = −0.0854). This meant that older adults in the CS-LI-HC had self-rated health scores 0.1079 points lower than those in the MS-MI-LC (a₁ = −0.1079). The relative direct effect was significant (c′₁ = −1.2290, *p* < 0.001), indicating that after excluding the mediating effect, older adults in the CS-LI-HC still had mental health scores 1.229 points lower than those in MS-MI-LC. The relative total effect was significant (c₁ = −1.3145, *p* < 0.001), with the effect size of the relative mediation effect (a₁b) being 6.50%.

**Figure 3:**
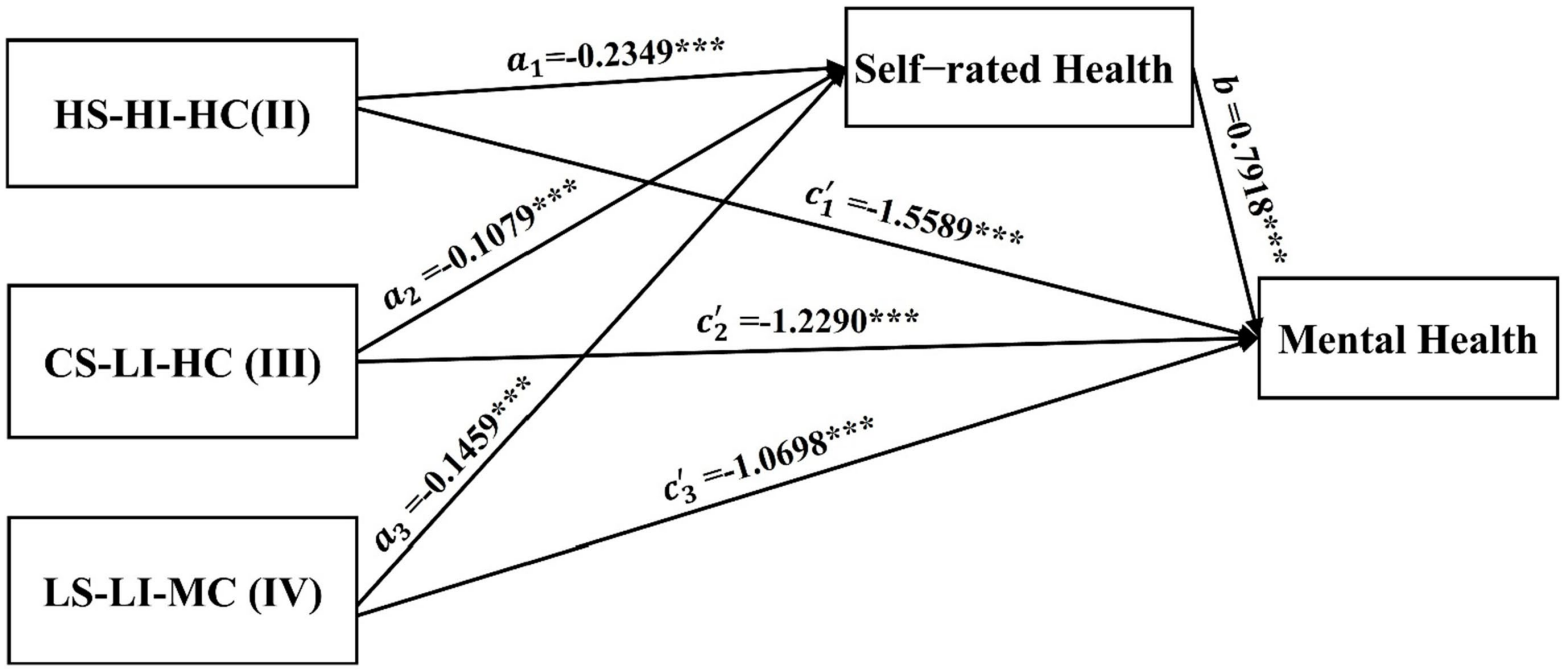
Indirect effect of self-rated health between intergenerational support and mental health (Reference: MS-MI-LC). Coefficients represent unstandardized effects. Higher scores on Self-Rated Health indicate poorer perceived health, and higher scores on Mental Health indicate greater depressive symptomatology (poorer mental health).

Using the MS-MI-LC as the reference, the 95% CI for the relative mediation effect of the LS-LI-MC was [−0.1891, −0.0440], excluding zero, indicating a significant relative mediation effect (a₁ = −0.1459, b = 0.7918, a₁b = −0.1155). This meant that older adults in the LS-LI-MC had self-rated health scores 0.1459 points lower than those in the MS-MI-LC (a₁ = −0.1459). The relative direct effect was significant (c′₁ = −1.0698, *p* < 0.001), indicating that after excluding the mediating effect, older adults in the LS-LI-MC still had mental health scores 1.0698 points lower than those in MS-MI-LC. The relative total effect was significant (c₁ = −1.1853, *p* < 0.001), with the effect size of the relative mediation effect (a₁b) being 9.74% (Table 7). Consistent with H3, these findings indicated that the mediating pathway of self-rated health between bidirectional intergenerational support and mental health in older adults held true (Figure 3).

**Table 7:** Mediating role of self-rated health between intergenerational support and mental health in older adults.

Item		Effect value	Boot SE	Boot 95% CI		Relative effect value
				Lower limit	Upper limit	
HS-HI-HC (II)	Total effect	−1.7449	—	—	—	—
	Indirect effect	−0.1860	0.0293	−0.2453	−0.1308	10.66%
	Direct effect	−1.5589	0.1185	−1.7912	−1.3266	89.34%
CS-LI-HC (III)	Total effect	−1.3145	—	—	—	—
	Indirect effect	−0.0854	0.0284	−0.1430	−0.0332	6.50%
	Direct effect	−1.2290	0.1232	−1.4705	−0.9876	93.50%
LS-LI-MC (IV)	Total effect	−1.1853	—	—	—	—
	Indirect effect	−0.1155	0.0376	−0.1890	−0.0446	9.74%
	Direct effect	−1.0698	0.1577	−1.3790	−0.7607	90.26%

Reference profile is MS-MI-LC; *p < 0.05, **p < 0.01, ***p < 0.001.

### 3.5 Moderated mediation: the role of social participation across support profiles

This study further incorporated social participation as a moderating variable and employed a bootstrap method with 5,000 resamples to test the moderated mediation effects of the multi-categorical independent variable. Table 8 presented the results of the moderated mediation analysis.

**Table 8:** Moderated mediation model results (*n* = 7,843).

Variables	Model 1(dependent variable: SRH)		Model 2(dependent variable: mental health)	
	β	t	β	t
Age	0.0574	3.0983**	0.1826	2.6787**
Gender	−0.0298	−1.5551	−0.1911	−2.709**
Marital status	0.0972	3.7767***	0.1919	2.0241*
Educational attainment	−0.0330	−4.1698***	−0.1884	−6.456***
Income	0.0123	1.0677	0.1554	3.6607***
Number of surviving children	0.0020	0.2309	−0.1604	−4.9283***
Number of social security programs	−0.0045	−0.2566	0.2002	3.1039**
Living arrangement	−0.0479	−1.2255	0.3025	2.1029*
Chronic status	0.1524	21.8858***	0.0802	3.0357**
Disability status	0.4586	11.3074***	0.5674	3.7665***
Place of residence	0.0134	0.6055	0.6953	8.5124***
HS-HI-HC (II)	−0.2334	−6.8707***	−1.4801	−12.338***
CS-LI-HC (III)	−0.0902	−2.6658**	−1.1781	−9.4588***
LS-LI-MC (IV)	−0.1016	−2.3535*	−0.9797	−6.1628***
SRH	—	—	0.7630	18.2704***
Social Participation	−0.1095	−3.1387**	−0.4273	−3.3286***
HS-HI-HC (II)* Social Participation	0.0099	0.2582	0.5111	3.6224***
CS-LI-HC (III)*Social Participation	0.0588	1.4842	0.6805	4.6649***
LS-LI-MC (IV)* Social Participation	−0.1767	−3.5633***	−0.0083	−0.0457
SRH* Social Participation	—	—	−0.2504	−5.4201***
R2	0.1323		0.1326	
F	66.2546***		59.7667***	

Reference profile is MS-MI-LC; *p < 0.05, **p < 0.01, ***p < 0.001.

First, compared with the MS-MI-LC, the indirect effect of the HS-HI-HC profile was significant at low social participation (W = −0.8455, *β* = −0.2259, SE = 0.0463, 95% CI = [−0.3209, −0.1378]), moderate social participation (W = 0, *β* = −0.1705, SE = 0.0283, 95% CI = [−0.2278, −0.1186]), and high social participation (W = +0.8455, *β* = −0.1185, SE = 0.0316, 95% CI = [−0.1844, −0.0613]), with the effect size decreasing as social participation increased. Second, compared with the MS-MI-LC, the indirect effect of the CS-LI-HC was significant at low social participation (*β* = −0.1364, SE = 0.0483, 95% CI = [−0.2336, −0.0426]) and moderate social participation (*β* = −0.0688, SE = 0.0281, 95% CI = [−0.1257, −0.0163]), but not significant at high social participation (*β* = −0.0223, SE = 0.0294, 95% CI = [−0.0833, 0.0336]). In contrast, the LS-LI-MC exhibited the opposite trend: the indirect effect was not significant at low social participation (*β* = 0.0466, SE = 0.0609, 95% CI = [−0.0712, 0.1649]), but became significant and progressively stronger at moderate social participation (*β* = −0.0775, SE = 0.0353, 95% CI = [−0.1489, −0.0103]) and high social participation (*β* = −0.1384, SE = 0.0390, 95% CI = [−0.2192, −0.0671]; Table 9).

**Table 9:** Indirect effect.

Types of effect	Profile	Moderating effect	Effect value	SE	Bootstrap 95%CI lower limit	Bootstrap 95%CI upper limit
Indirect effect	HS-HI-HC (II)	M-1SD	−0.2259	0.0463	−0.3209	−0.1378
		M	−0.1705	0.0283	−0.2278	−0.1186
		M + 1SD	−0.1185	0.0316	−0.1844	−0.0613
	CS-LI-HC (III)	M-1SD	−0.1364	0.0483	−0.2336	−0.0426
		M	−0.0688	0.0281	−0.1257	−0.0163
		M + 1SD	−0.0223	0.0294	−0.0833	0.0336
	LS-LI-MC (IV)	M-1SD	0.0466	0.0609	−0.0712	0.1649
		M	−0.0775	0.0353	−0.1489	−0.0103
		M + 1SD	−0.1384	0.039	−0.2192	−0.0671

Effects presented are unstandardized coefficients. Reference profile is MS-MI-LC; *p < 0.05, **p < 0.01, ***p < 0.001.

**Figure 4:**
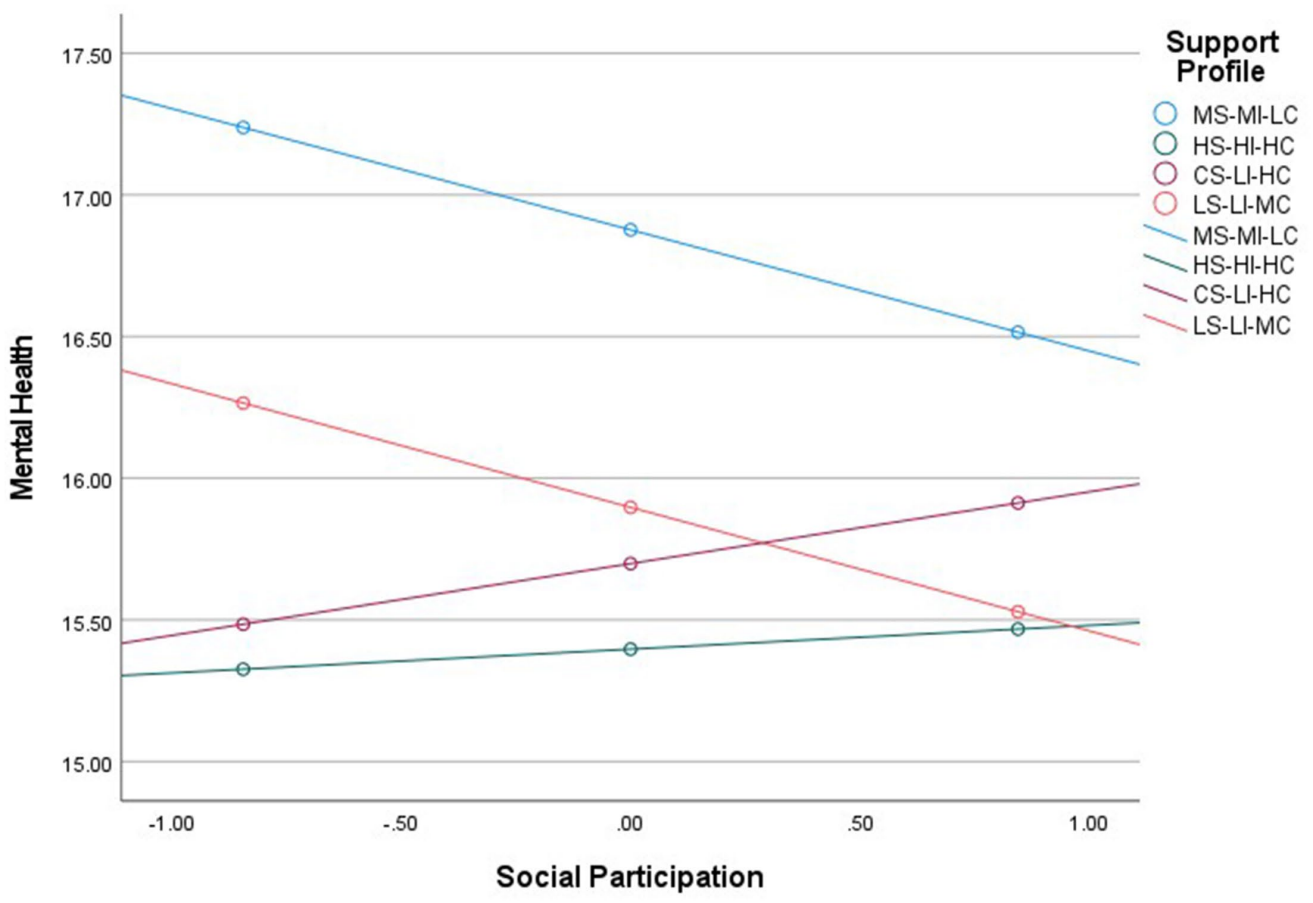
The moderating effect of social participation on the association between intergenerational support profiles and mental health.

Subsequent analysis of direct effects revealed that, compared with the MS-MI-LC, the HS-HI-HC had significant direct effects on mental health at low social participation (*β* = −1.9122, SE = 0.1536, 95% CI [−2.2132, −1.6112]), medium social participation (*β* = −1.4801, SE = 0.1200, 95% CI [−1.7153, −1.2449]), and high social participation (*β* = −1.0480, SE = 0.1835, 95% CI [−1.4076, −0.6884]), with the effect size decreasing as social participation increased. The CS-LI-HC showed a similar trend: its direct effects were significant at low social participation (*β* = −1.7534, SE = 0.1656, 95% CI [−2.0781, −1.4287]), medium social participation (*β* = −1.1781, SE = 0.1246, 95% CI [−1.4223, −0.9339]), and high social participation (*β* = −0.6028, SE = 0.1844, 95% CI [−0.9643, −0.2413]), with the effects likewise declining. In contrast, the LS-LI-MC exhibited relatively stable and significant direct effects at low social participation (*β* = −0.9726, SE = 0.2170, 95% CI [−1.3980, −0.5473]), medium social participation (*β* = −0.9797, SE = 0.1590, 95% CI [−1.2913, −0.6681]), and high social participation (β = −0.9867, SE = 0.2261, 95% CI [−1.4300, −0.5435]; Table 10). The interaction plot visualizing these conditional direct effects is presented in Figure 4.

**Table 10:** Direct effect.

Types of effect	Profile	Moderating effect	Effect value	SE	t
Direct effect	HS-HI-HC (II)	M-1SD	−1.9122	0.1536	−12.4518***
		M	−1.4801	0.12	−12.338***
		M + 1SD	−1.0480	0.1835	−5.7126***
	CS-LI-HC (III)	M-1SD	−1.7534	0.1656	−10.5857***
		M	−1.1781	0.1246	−9.4588***
		M + 1SD	−0.6028	0.1844	−3.2688***
	LS-LI-MC (IV)	M-1SD	−0.9726	0.217	−4.4825***
		M	−0.9797	0.159	−6.1628***
		M + 1SD	−0.9867	0.2261	−4.364***

Effects presented are unstandardized coefficients. Reference profile is MS-MI-LC; *p < 0.05, **p < 0.01, ***p < 0.001.

In line with H4, the above analysis indicated that social participation moderates the pathways through which different profiles of bidirectional intergenerational support affect mental health. For the HS-HI-HC and CS-LI-HC, both indirect and direct effects weakened as social participation increased; in contrast, the LS-LI-MC exhibited indirect effects that strengthened with higher social participation and relatively stable direct effects.

## 4 Discussion

Drawing on intergenerational solidarity theory and social support theory, this study utilized data from the 2020 CLASS survey to examine profiles of bidirectional intergenerational support in China and their associations with older adults’ mental health. By conducting latent profile analysis across three core dimensions—economic, emotional, and caregiving support—we identified four distinct support profiles. We further explored how these profiles differentially affected mental health and tested the mediating role of self-rated health and the moderating effect of social participation.

First, four distinct profiles of bidirectional intergenerational support were identified in this study: MS-MI-LC, HS-HI-HC, CS-LI-HC, and LS-LI-MC. The latent profile analysis was conducted on a nationally representative dataset comprising 7,843 respondents from the 2020 wave of the China Longitudinal Aging Social Survey (CLASS), ensuring robust generalizability. The MS-MI-LC profile accounted for 10.37% of the sample, a proportion consistent with previous studies (58). In this group, although older adults and their children maintained relatively frequent contact and mutual support, the level of emotional closeness was notably low. This may be attributed to children merely fulfilling their support obligations while neglecting their parents’ emotional needs. Such families were relatively uncommon in the sample. The HS-HI-HC profile represented 47.02% of the sample, making it the predominant pattern of intergenerational support in China. In this group, older adults and their children engaged in reciprocal support not only economically but also through caregiving and emotional bonds. This finding is consistent with prior research that highlights the multidimensional reciprocity typical of Chinese family relationships (20, 59). The CS-LI-HC profile accounted for 33.46% of the sample. In this group, interaction frequency was relatively low—potentially due to physical distance or time constraints—yet children provided substantial economic support, and emotional closeness remained high. Previous studies have shown that many older adults who do not co-reside with their children still receive adequate support, particularly when their children are economically well-off, which enables elders to live independently and maintain good psychological well-being (21). The LS-LI-MC profile was the least common, comprising 9.16% of the sample. Older adults in this category received relatively low levels of both economic and caregiving support, yet maintained a moderate level of emotional closeness with their children. This may reflect a pattern of mutual independence, possibly due to the older adults’ stable sources of income such as pensions and social insurance, which allow them to maintain economic autonomy and avoid becoming a burden to their children (60). Overall, the HS-HI-HC profile is the dominant pattern of intergenerational support in China, characterized by high levels of emotional intimacy and mutual assistance. This pattern is deeply rooted in Confucian values (61), which emphasize filial piety and the obligation of children to support their aging parents through economic provision and daily care. At the same time, older adults often provide reciprocal support—both materially and emotionally—when their children face economic or caregiving burdens (21).

Second, our findings confirm Hypotheses 1 and 2, demonstrating that all profiles of bidirectional intergenerational support exert positive effects on mental health, though the magnitude of these effects varies significantly. Among them, the HS-HI-HC profile is most beneficial to the psychological well-being of older adults. Individuals in this group receive reciprocal support from their children across economic, emotional, and caregiving domains, fulfilling their multidimensional needs. In the Chinese cultural context, such reciprocity is also viewed as an expression of filial piety. These findings align with prior studies suggesting that “reciprocal” family relationships are particularly effective in fostering older adults’ sense of positivity and self-worth, thereby improving their mental health (21). When older adults not only receive care and emotional comfort from their children but also contribute to household affairs or grandchild caregiving, they are more likely to experience psychological fulfillment and reduced levels of depression (62, 63). In contrast, older adults in the MS-MI-LC profile exhibited the highest depression scores, indicating the poorest mental health among all groups. Although these individuals receive moderate levels of economic and caregiving support, the markedly low emotional closeness may contribute to psychological strain or feelings of loneliness. In such cases, children may fulfill their caregiving duties while neglecting their parents’ emotional needs, potentially undermining the mental well-being of older adults. This study provides empirical support for the hypothesis that the absence of emotional support may offset or weaken the protective effects of economic and caregiving support on older adults’ mental health. Older adults in the CS-LI-HC group reported the second-best mental health outcomes, slightly better than those in the LS-LI-MC group. For this subgroup, limited caregiving and interaction—potentially due to geographical distance or time constraints—did not appear to compromise emotional closeness. Children still served as the primary economic providers and offered sufficient emotional reassurance, which helped maintain strong relational bonds and supported older adults’ psychological well-being (64, 65). This finding is consistent with previous literature, suggesting that older adults tend to prioritize the quality rather than the frequency or quantity of intergenerational support (66, 67). When high levels of economic or caregiving support cannot be sustained—and emotional closeness is also lacking—older adults’ mental health is likely to deteriorate. Ideally, bidirectional intergenerational support should be characterized by mutual reciprocity in both material and emotional domains. However, when full reciprocity is not feasible, maintaining emotional intimacy and ensuring the provision of emotional reassurance remain critical strategies for safeguarding the psychological well-being of aging parents (68, 69).

Third, this study found that, relative to the MS-MI-LC profile, self-rated health significantly mediated the relationship between mental health and the other three support profiles—namely, HS-HI-HC, CS-LI-HC, and LS-LI-MC. These results support Hypothesis 3. Prior research has demonstrated that intergenerational support positively affects self-rated health, consistent with social support theory (70), which posits that supportive exchanges can enhance older adults’ functional capacity and psychological resilience (71, 72). Receiving support from children can effectively alleviate depressive symptoms among older adults (73). For instance, with assistance from their children, older adults are more likely to seek timely medical attention when experiencing physical discomfort (74). Furthermore, older adults who provide assistance to their children may also experience improved self-rated health, which subsequently manifests in better psychological outcomes, such as reduced depression and enhanced life satisfaction. This may be because bidirectional intergenerational support simultaneously meets the material and health-related needs of older adults while reinforcing their sense of self-worth. When older adults provide support to their children, they may experience a stronger sense of usefulness and autonomy. This realization of personal value can increase their confidence in managing their health, thereby alleviating depressive symptoms and improving overall mental well-being (73).

Fourth, this study found that social participation moderated the association between bidirectional intergenerational support and older adults’ mental health, partially supporting Hypothesis 4. Compared with the MS-MI-LC profile (Moderate Support-Moderate Interaction-Low Closeness; defined at first use), stronger-support profiles showed weaker direct and indirect effects as social participation increased, whereas lower-support profiles showed stronger effects, which means the protective effects of intergenerational support on mental health diminish as social participation increases. Framed by social support theory and resource substitution, two mechanisms are plausible. First, when family-based emotional/economic/caregiving support is already abundant, additional community ties add redundant resources, producing diminishing marginal returns and attenuating associations. Second, when family support is scarce, social participation supplies functionally equivalent belonging, identity, and companionship that buffers stress and amplifies the (indirect) benefits of limited support (buffering hypothesis). These patterns are consistent with prior evidence that social participation yields stronger positive effects on life satisfaction for Chinese older adults lacking family companionship (75). When the primary family support is lacking, external social participation becomes a critical compensatory resource. It provides a sense of belonging, self-actualization, and social connections that buffers the individual from the potential negative psychological consequences of insufficient family support, which reflects the synergistic effects of family-based support and community-level intergenerational interaction on the mental health of older adults (28, 46).

Building on latent profile analysis of bidirectional intergenerational support profiles, this study examined their effects on mental health in older adults, while also revealing the mediating role of self-rated health and the moderating role of social participation. These findings extend the explanatory framework for understanding psychological well-being in later life, highlight the heterogeneity of intergenerational support, and resonate with Confucian filial piety—offering strong explanatory power in societies characterized by strong family-oriented cultural traditions.

This study has several limitations. First, key indicators were measured with simplified instruments. This study followed the common practices employed in large-scale surveys, using a short form of the CES-D to measure mental health, while self-rated health was measured with a single item and social participation with a formative index based on activity counts. However, it may not capture construct multidimensionality. Specifically, the single-item health measure may lack the reliability of multi-item scales, and activity counts fail to reflect the quality of participation or subjective experience. This reduced reliability and construct coverage can introduce attenuation bias. Consequently, the modest effect sizes observed in this study should be interpreted with caution, as they are likely conservative estimates and may underestimate the true strength of the relationships. This potentially underestimates the actual theoretical and practical significance of our findings. Future studies should consider incorporating multi-source data to mitigate common source bias and improve objectivity. For instance, matched dyadic surveys with older adults and children could provide a multi-informant measure of the intergenerational support variables. Furthermore, wearable device data could offer objective behavioral proxies for constructs currently self-reported, such as social participation (for instance, time out-of-home) and health status (for instance, sleep quality, physical activity levels), which are closely correlated with mental health. Second, the “classify-then-analyze” approach treated latent profile membership as perfectly known when entered into the mediation models, which does not propagate classification uncertainty from the LPA step into the mediation step. This may lead to biased estimates of the indirect and conditional effects. This study chose the pragmatic approach to leverage the interpretability of the PROCESS macro, relying on high classification quality (Entropy = 0.895) to suggest that this bias is minimized, but the findings should be interpreted with this in mind. Third, we did not account for the complex survey design of CLASS (for instance, clustering and weights) and relied on listwise deletion for missing data. While the unweighted models are suitable for exploring theoretical relationships, the prevalence estimates (such as 47.02% for the HS-HI-HC profile) may be distorted. The use of listwise deletion assumes data are missing completely at random, which is a strong assumption. These factors limit the generalizability of the descriptive statistics and may affect the precision. Fourth, due to the cross-sectional nature of the study design, the findings reflect associations, and causal inferences cannot be firmly established. Future research should adopt longitudinal designs to explore causal directions among the variables. Finally, as all participants were drawn from China, our findings are likely culturally-bound by the context of collectivism and filial piety. In more individualistic western cultures, where autonomy and independence in later life are valued, intergenerational support may differ significantly. The “high support” profiles may be less prevalent, and some older adults could construe intensive family help as dependency, dampening mental-health benefits. Under such conditions, the substitution role of social participation may be stronger. Therefore, cross-cultural research in such contrasting contexts is needed not to simply replicate the findings, but to test these specific hypotheses and thereby validate the cultural boundary conditions of our conclusions.

## 5 Conclusion

In summary, this study yields several key conclusions. First, four distinct profiles of bidirectional intergenerational support were identified in China: MS-MI-LC, HS-HI-HC, CS-LI-HC, and LS-LI-MC. The HS-HI-HC profile was the most prevalent, indicating that reciprocal and emotionally close intergenerational relationships remain the dominant form of support in contemporary China. This finding further affirms and extends the applicability of intergenerational solidarity theory within the Chinese cultural context. Second, there were significant differences in the effects of support profiles on older adults’ mental health. Compared to the reference group, the HS-HI-HC profile was associated with significantly lower depressive symptoms. The CS-LI-HC and LS-LI-MC profiles had intermediate protective effects. These findings suggest that emotional closeness plays a more critical role in promoting mental health than economic or caregiving support alone. In the absence of emotional connection, even adequate material support may not sufficiently protect mental health. This highlights the importance of incorporating emotional bonding and interaction into intervention policies aimed at enhancing the well-being of older adults. Third, self-rated health partially mediated the association between intergenerational support and mental health. Compared to the MS-MI-LC group, the other three profiles were associated with improved self-rated health, which in turn was linked to reduced depressive symptoms. These results underscore the value of self-rated health as a key entry point for mental health interventions among older adults. Health authorities and related sectors should prioritize initiatives that enhance older adults’ health awareness and engage both older adults and their children in promoting health perceptions to achieve positive psychological outcomes. Fourth, social participation moderated the mediation pathway, with effects varying across support profiles. Among those with high levels of intergenerational support, the protective effect diminished as social participation increased, suggesting a compensatory effect of external support. Conversely, for those with low intergenerational support, greater social participation significantly enhanced the positive impact of self-rated health on mental well-being. This reflects the synergistic interaction between intergenerational and social support systems. These findings suggest that intervention strategies should be tailored to differences in intergenerational support profiles among older adults. For those with low support, programs such as community volunteering or elder-focused social activities can help compensate for the absence of family-based support. For those with strong intergenerational support, efforts should focus on maintaining the quality of family relationships while also encouraging moderate social participation to diversify sources of psychological support. Thus, this study highlights the importance of enhancing familial closeness and support, as well as promoting social engagement, in improving the mental health of older adults. These findings not only enrich the existing literature on intergenerational support and mental health but also offer practical implications for policy design. Given the cross-sectional design and the modest effect sizes observed, further longitudinal and experimental research is warranted to validate and extend these findings.

## Data Availability

The datasets generated and/or analyzed during the current study are available in the China Longitudinal Aging Social Survey (CLASS) repository, http://class.ruc.edu.cn.
