# Supplementary material for: Bidirectional intergenerational support and mental health in older adults based on latent profile analysis: a moderated mediation model
Source: Front Public Health. 2025 Dec 10;13:1685701. doi: 10.3389/fpubh.2025.1685701 (PMC12727621; doi:10.3389/fpubh.2025.1685701)
Supplement: Supplementary file 1 [file Table_1.DOCX]

Supplementary Material

# Supplementary Tables

**Supplementary Table 1**. Correlation matrix for the total sample

| Variables | 1 | 2 | 3 | 4 | 5 | 6 | 7 | 8 | 9 | 10 | 11 | 12 | 13 | 14 | 15 | 16 | 17 | 18 | 19 | 20 | 21 |
| --- | --- | --- | --- | --- | --- | --- | --- | --- | --- | --- | --- | --- | --- | --- | --- | --- | --- | --- | --- | --- | --- |
| 1.Child-to-Parent Economic Support | 1 | — | — | — | — | — | — | — | — | — | — | — | — | — | — | — | — | — | — | — | — |
| 2. Parent-to-Child Economic Support | .322** | 1 | — | — | — | — | — | — | — | — | — | — | — | — | — | — | — | — | — | — | — |
| 3.Frequency of Contact | -.149** | -.165** | 1 | — | — | — | — | — | — | — | — | — | — | — | — | — | — | — | — | — | — |
| 4.Frequency of In-Person Contact | -.122** | -.197** | .571** | 1 | — | — | — | — | — | — | — | — | — | — | — | — | — | — | — | — | — |
| 5.Parent–Child Emotional Closeness | .087** | .039** | -.182** | -.081** | 1 | — | — | — | — | — | — | — | — | — | — | — | — | — | — | — | — |
| 6.Frequency of children helping parents with housework | -.125** | -.199** | .419** | .570** | -.046** | 1 | — | — | — | — | — | — | — | — | — | — | — | — | — | — | — |
| 7.Frequency of parents helping children with housework | -.063** | -.239** | .212** | .335** | .029* | .391** | 1 | — | — | — | — | — | — | — | — | — | — | — | — | — | — |
| 8.Age | -.068** | -.139** | .082** | 0.011 | -.059** | 0.014 | .131** | 1 | — | — | — | — | — | — | — | — | — | — | — | — | — |
| 9.Gender | -0.022 | -0.014 | .053** | .060** | -0.01 | .036** | .049** | 0.006 | 1 | — | — | — | — | — | — | — | — | — | — | — | — |
| 10.Marital status | -.056** | -.083** | -0.013 | -.057** | -.052** | -.049** | -0.018 | .233** | -.171** | 1 | — | — | — | — | — | — | — | — | — | — | — |
| 11.Educational attainment | .180** | .229** | -.159** | -.085** | .074** | -.083** | -.068** | -.227** | .145** | -.163** | 1 | — | — | — | — | — | — | — | — | — | — |
| 12.Income | .261** | .264** | -.284** | -.295** | -0.014 | -.223** | -.152** | -0.009 | -.030** | -.062** | .291** | 1 | — | — | — | — | — | — | — | — | — |
| 13.Number of surviving children | -.164** | -.262** | .303** | .241** | -.068** | .258** | .217** | .359** | -.034** | .115** | -.296** | -.210** | 1 | — | — | — | — | — | — | — | — |
| 14.Number of social security programs | -0.017 | -.079** | .043** | -0.004 | -.031** | 0.001 | .067** | .601** | 0.018 | .114** | -.098** | .046** | .176** | 1 | — | — | — | — | — | — | — |
| 15.Living arrangement | -.041** | -.089** | .104** | .114** | -.050** | .085** | .084** | .082** | -.076** | .528** | -.085** | -.239** | .052** | .047** | 1 | — | — | — | — | — | — |
| 16.Chronic status | .072** | .078** | 0.006 | -.049** | -0.015 | -0.012 | -0.016 | .188** | -.076** | .084** | -.081** | .163** | .086** | .190** | -.027* | 1 | — | — | — | — | — |
| 17.Disability status | .031** | 0.005 | -.067** | -.093** | -0.009 | -.146** | -.029** | .185** | 0.01 | .091** | -.051** | .043** | .036** | .107** | -.040** | .104** | 1 | — | — | — | — |
| 18.SRH | .044** | -.037** | .085** | 0.017 | -.077** | -0.015 | -0.009 | .161** | -.049** | .101** | -.103** | .039** | .082** | .109** | 0.014 | .281** | .169** | 1 | — | — | — |
| 19.Social participation | -.044** | -.026* | .045** | .085** | .049** | .104** | -.054** | -.195** | .050** | -.070** | -.023* | -.136** | .024* | -.137** | -0.016 | -.078** | -.087** | -.135** | 1 | — | — |
| 20.Place of residence | -.168** | -.205** | .192** | .179** | -.023* | .121** | .022* | .037** | .056** | 0.021 | -.338** | -.436** | .255** | -0.001 | 0.019 | -.023* | 0.019 | 0.009 | .207** | 1 | — |
| 21.Mental health | -.102** | -.114** | .060** | .027* | -.159** | 0.017 | -.066** | .129** | -.049** | .097** | -.145** | -0.003 | .047** | .104** | .050** | .126** | .100** | .266** | -.026* | .110** | 1 |
| Mean | 4.2806 | 2.2416 | 2.0758 | 2.3989 | 2.8541 | 2.9863 | 4.034 | 1.6826 | 0.5 | 1.24 | 3.03 | 7.4097 | 2.42 | 1.4964 | 0.094 | 1.6939 | 0.06 | 2.58 | 0.9819 | 0.43 | 15.7342 |
| SD | 1.65509 | 1.64032 | 0.70104 | 0.87925 | 0.31653 | 1.1974 | 1.28969 | 0.69493 | 0.5 | 0.447 | 1.339 | 0.97606 | 1.235 | 0.66622 | 0.2918 | 1.39414 | 0.235 | 0.878 | 0.84546 | 0.496 | 3.22933 |
| Max | 9.5 | 7.33 | 4.33 | 5 | 3 | 5 | 5 | 3 | 1 | 4 | 7 | 11.51 | 9 | 6 | 1 | 13 | 1 | 5 | 3 | 1 | 25 |
| Min | 1 | 1 | 1 | 1 | 1.8 | 1 | 1 | 1 | 0 | 1 | 1 | 3 | 1 | 0 | 0 | 0 | 0 | 1 | 0 | 0 | 9 |
